# Light-responsive and corrosion-resistant gas valve with non-thermal effective liquid-gating positional flow control

**DOI:** 10.1038/s41377-021-00568-9

**Published:** 2021-06-16

**Authors:** Baiyi Chen, Rongrong Zhang, Yaqi Hou, Jian Zhang, Shiyan Chen, Yuhang Han, Xinyu Chen, Xu Hou

**Affiliations:** 1 State Key Laboratory of Physical Chemistry of Solid Surfaces, College of Chemistry and Chemical Engineering, Xiamen University, Xiamen, 361005 China; 2 Collaborative Innovation Centre of Chemistry for Energy Materials, Xiamen University, Xiamen, 361005 China; 3 Department of Physics, Research Institute for Biomimetics and Soft Matter, Fujian Provincial Key Laboratory for Soft Functional Materials Research, Jiujiang Research Institute, College of Physical Science and Technology, Xiamen University, Xiamen, 361005 China; 4 Tan Kah Kee Innovation Laboratory, Xiamen, 361102 China

**Keywords:** Physics, Optical techniques

## Abstract

Safe and precise control of gas flow is one of the key factors to many physical and chemical processes, such as degassing, natural gas transportation, and gas sensor. In practical application, it is essential for the gas-involved physicochemical process to keep everything under control and safe, which significantly relies on the controllability, safety, and stability of their valves. Here we show a light-responsive and corrosion-resistant gas valve with non-thermal effective liquid-gating positional flow control under a constant pressure by incorporating dynamic gating liquid with light responsiveness of solid porous substrate. Our experimental and theoretical analysis reveal that the photoisomerization of azobenzene-based molecular photoswitches on the porous substrate enabled the gas valve to possess a light-responsive and reversible variation of substantial critical pressure of non-thermal effective gas flow switch. Moreover, the chemically inert gating liquid prevented the solid substrate from corrosion and, by combining with the high spatiotemporal resolution of light, the gas valve realizes a precisely positional open and close under a steady-state pressure. The application demonstrations in our results show the potentials of the new gas valve for bringing opportunities to many applications, such as gas-involved reaction control in microfluidics, soft actuators, and beyond.

## Abstract (web summary)

A light-responsive and corrosion resistant gas valve with non-thermal effective liquid gating positional flow control under a constant pressure by incorporating dynamic gating liquid with light-responsiveness of solid porous substrate.

## Graphical abstract

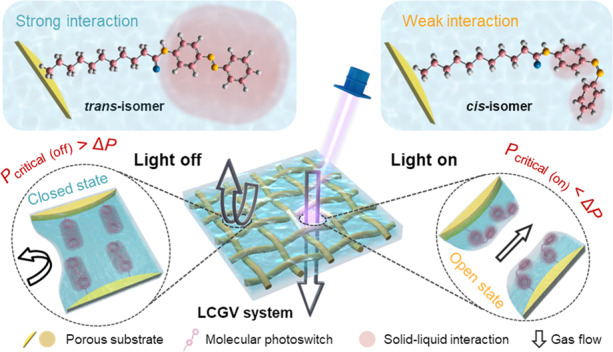

The capability to precisely contactless control the gas flow in a safe and steady way is essential to gas valves due to its guarantee of the utility and stability for various applications such as gas mining, fermentation, multiphase separation, soft actuators and robots, and many other fields^1–5^. However, its improvement remains challenging, especially the stable position-specific gas delivery, which normally relies on a sophisticated automation control system based on the external input^6–8^. Recently, the liquid-gating technology with utilizing the capillary-stabilized liquid as a dynamic and reconfigurable gate to govern the fluid transport^9–12^ is put forward and attracts widespread attention as an emerging alternative of conventional fluid control valve for manifold applications in controllable and stable physical and chemical processing such as multiphase separation and microfluidic reactors^13–16^. Based on a rational interfacial design, the liquid-filling pores can be completely closed by liquid sealing and dynamically forced open a liquid-lined pathway under pressure, in which the liquid gate exerts a flexible and stable interfacial switching performance for transport fluid and prevents it from contacting the solid porous substrate, thereby showing inherently manageable and corrosion-resistant benefits to break the limitations of conventional mechanical fluid control valves^17–19^.

However, liquid gating technology is still at an early stage and there are many challenges to overcome for its development, such as the exploitation of contactless position-specific fluid control responding to the diversified external stimuli such as thermal and light, which can make up for the difficulty to deal with the complex fluid control scenarios, owing to its simplex pressure-driven mechanism. With regard to the diversified field controls, the light control, with inherent merits of flexibly contactless interaction and high spatiotemporal resolution^20,21^, mainly includes the photophysical and photochemical mechanisms, which possess their own advantages and corresponding application scenarios. For instance, the photophysical control, generally accompanied by the changes in other physics field such as thermal, can be used to conveniently realize the fluid control in the complicated application scenarios that require multiphysics synergy^22–24^. However, many delivery systems require stable and interference-free single-field stimulus to realize the safe and precise fluid control in a steady surrounding, such as the compressed natural gas transportation. Thus, the photochemical control with single local physical stimulus should be further considered, to ensure a stable and precise contactless fluid control without other physical interference^25,26^.

Here we show a strategy to develop a photochemical-responsive and corrosion-resistant gas valve (light-responsive and corrosion-resistant gas valve, LCGV) with non-thermal effective liquid-gating positional flow control. To advance a rational light-responsive solid–liquid interfacial design, the photoisomerization of azobenzene-based molecular photoswitches was employed to endow the interface of porous substrate and gating liquid with controllable solid–liquid interaction, which further induced a thermal interference-free, light-regulated, and reversible variation of substantial critical pressures of transport gas with this LCGV system. Before ultraviolet (UV) irradiation, the *trans*-formed molecular photoswitches on solid porous substrate possess a stronger interaction with the nonpolar gating liquid and a small gas pressure difference between both sides of LCGV system cannot open the pores, resulting in a closed state for gas transport with this gas valve. Whereas under UV irradiation, the molecular photoswitches undergo a *trans*-to-*cis* photoisomerization with a significant decrease of solid–liquid interaction, which reduces the substantial critical pressure of the LCGV system and exhibits an open state for gas transport. Meanwhile, the LCGV system shows a good corrosion resistance as a result of gating liquid preventing the corrosive gas from contacting with the porous substrate. In addition, owing to the athermal interaction and high spatiotemporal resolution of UV light, this light-responsive gas valve is endowed with a precisely non-thermal effective positional gas flow control, which can be used for position-specific reaction as a microfluidic reactor. Consequently, this functionally integrated liquid-gating gas valve introduces a new paradigm to provide more possibilities for the liquid gating strategy, meet the needs of diversified application scenarios, and further promote the development and breakthrough of gas control valves.

The affinity between solid porous substrate and functional gating liquid is one of the key factors to determine the pressure threshold of a liquid gating membrane^9,10^. Therefore, the chemical composition of a solid substrate surface is closely related to the working pressure range of the a liquid gating system. As shown in Fig. 1, by incorporating the dynamic nature of liquids with photochemical-responsive properties of porous substrate achieving through molecular photoswitches grafting, the light-responsive liquid gating system is formed. As the most widely studied molecular photoswitch, azobenzene and its derivatives can be triggered rapidly by athermal UV light (365~400 nm) to achieve a reversible *trans*-to-*cis* photoisomerization, which would significantly change its molecular polarity, thereby changing the solid–liquid interaction between gating liquid and photoswitches grafted solid porous substrate (Fig. 1a). Before UV irradiation (light off, top), the azobenzene-based molecular photoswitches on porous substrate show a *trans* form and a strong interaction with the nonpolar gating liquid. When the pressure difference on both sides of the porous substrate Δ*P* is lower than the substantial critical pressure of transport gas *P*_critical (off)_, the LCGV system is in the closed state. Whereas under UV irradiation (light on, bottom), the azobenzene-based molecular photoswitches undergo a *trans*-to-*cis* photoisomerization, which leads to a robust increase in molecular polarity and weak interaction with the nonpolar gating liquid. If Δ*P* higher than the substantial critical pressure of transport gas *P*_critical (on)_, gas will force open the liquid gate and LCGV system is open. After removing UV irradiation, the photoswitches reverse to *trans* isomer rapidly driven by mild indoor visible light^27^, which brings LCGV system back to the initial closed state.

**Fig. 1:**
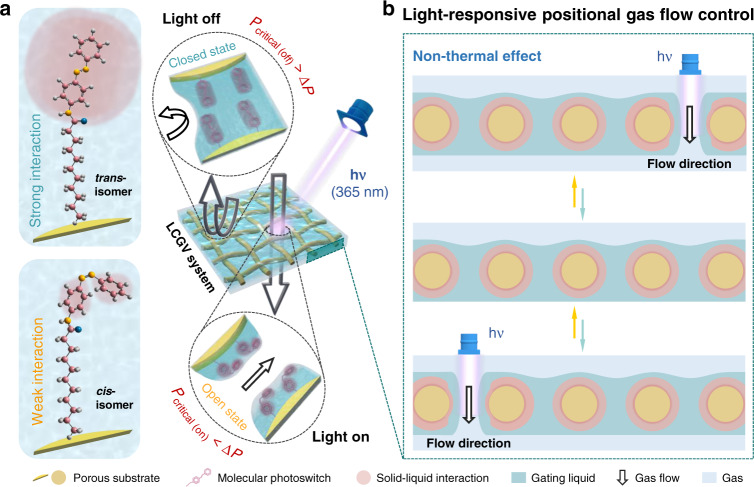
Working principle and non-thermal effective positional gas flow control of the light-responsive and corrosion-resistant gas valve (LCGV). **a** Schematic diagram of the closed and open states of LCGV system. The reversible photoisomerization of molecular photoswitches induces a change in the solid–liquid interaction between the solid porous substrate and functional gating liquid^36^, which further causes the variation of the substantial gas critical pressures of the LCGV system. **b** Gas flow can be precisely navigated and dynamically controlled without thermal interference by LCGV system due to the athermal interaction and high spatiotemporal resolution of UV light

Based on the combination of the above phenomena and the free localizability, athermal interaction, and contactless and fast response of UV light, the LCGV system can be employed as a non-thermal effective light-induced gas valve with dynamic and regulatory gas flow control in selected area (Fig. 1b). Localized UV irradiation triggers the photoisomerization of molecular photoswitches and the transmembrane critical pressure of gas rapidly reduces in the irradiation area, where the gas is allowed to locally force open the liquid-filling pores but remain closed in the other area under a constant pressure (Δ*P*). By repositioning the light source, the gating liquid reconfigures and closes the substrate pores in previous irradiation area, and the transport gas starts to penetrate through the LCGV system in the light regulatory area driven by constant pressure. Thus, the photoisomerization of azobenzene-based molecular photoswitches endows the liquid gating system with light-responsive properties including high spatiotemporal resolution, defect-free reversibility, and remote controllability, thereby realizing the precise navigation and dynamic regulation of gas flow without thermal interference by the LCGV system.

The stainless-steel membrane (SSM) with robust mechanical property and optical inertness is used to construct the LCGV system. After Au-ion sputtering (Au/SSM) and molecular photoswitches modification (Azo/SSM) (Supplementary Fig. S1), the metallic membrane is endowed with light-responsive property. By simply infusing the gating liquid in this light-responsive porous membrane, the LCGV system is created (Fig. 2a). Infrared (IR) spectra have demonstrated the successful synthesis of azobenzene-based molecular photoswitch (iii) (Fig. 2b), which is obtained by one-step substitution from flexible intermediate (i) and azobenzene derivatives (ii) (Supplementary S2). Under the UV and visible light-alternating stimuli, UV-visible absorption analysis indicates this molecular photoswitch undergoes an expected *trans*-to-*cis* photoisomerization and *trans*-to-*cis* reversion (Fig. 2c and Supplementary Fig. S2), where in the peaks at around 365 and 435 nm could be assigned to the *π*–π* and *n*–π* adsorptions, respectively^28^. The reversible photoisomerization causes a variation in the molecular polarity of the photoswitches, which further changes the surface energy (SE) and wettability of its modification surface^29^. Thus, under light stimuli, the liquid droplets would present controllable wetting behavior on the light-responsive surface (Supplementary Fig. S3).

**Fig. 2:**
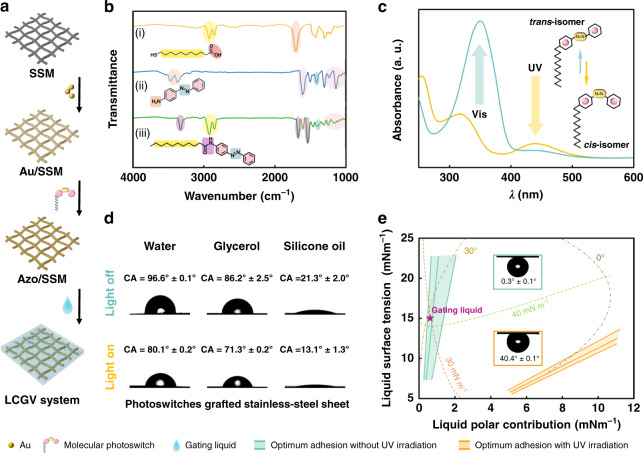
Design, evaluation and adhesion mechanism of LCGV system. SSM, Au/SSM, and Azo/SSM represent the stainless-steel membrane, Au-ion sputtered stainless-steel membrane, and azobenzene-based molecular photoswitches grafted stainless-steel membrane. **a** Interfacial design and preparation of the LCGV system. **b** Infrared spectra demonstrate the successful synthesis of azobenzene-based molecular photoswitch. **c** UV-vis absorption spectra of the azobenzene-based molecular photoswitch with *trans* and *cis* forms. Inset shows the photoisomerization process of the molecular photoswitch. **d** The wettability of different liquids on photoswitches grafted stainless-steel sheet with and without UV irradiation. **e** The solid–liquid interfacial adhesion mechanism of the LCGV system. Insets show the bubble contact angles on photoswitches grafted stainless-steel sheet immersing in gating liquid with and without UV irradiation

As shown in Fig. 2d, the investigation on the light-regulated wettability of different liquids on the molecular photoswitches grafted surface is conducted. The water, glycerol, and silicone oil droplets with different surface tension (SFT) reveal varying degrees of contact angle (CA) changes under light stimuli, indicating the solid–liquid configuration is also a key factor for interfacial energy variations in the light-responsive system. With respect to LCGV system, the solid–liquid interfacial property is the utmost critical factor to the system feasibility and stability, which directly affects the adhesion behavior between functional gating liquid and solid porous substrate, and thus the gating performance during gas transport. To satisfy the interfacial design for the LCGV system, the work of adhesion (*W*_A_) analysis according to Owens–Wendt–Rabel–Kälble (OWRK) method is used for the selection of solid–liquid configurations (Supplementary S4 and Fig. S4)^30–32^. Among all the alternative configurations, the nonpolar functional liquid of Krytox® 103 shows a providential adhesion performance with the chosen light-responsive surface, which is ultimately utilized as the gating liquid for the LCGV system (Fig. 2e). Before UV irradiation, Krytox® 103 exerts an excellent adhesive property (being in the corridor for optimum adhesion without UV irradiation and showing the *W*_a_ value of 41.0 mN m^−1^) and spreading behavior (being in 0° wetting envelop) on the light-responsive surface, due to the strong interaction between nonpolar liquid and *trans*-formed molecular photoswitches. Whereas under UV irradiation, the azobenzene-based molecular photoswitches undergo a conformational change to *cis*-isomer, resulting in a dramatical increase in solid membrane surface polarity and a lower solid–liquid adhesion (being out the corridor for optimum adhesion with UV irradiation and showing the *W*_a_ value of 29.2 mN m^−1^). Subsequently, a light-regulated solid–liquid adhesion interface corresponding to the solid surface polarity changes is achieved.

By utilizing the solid–liquid adhesive mechanism for the confined pores of porous substrate, the gating performance of LCGV system can be well explained. In this liquid gating system, critical pressure thresholds *P*_critical (gas)_, the forces required for gas to overcome the capillary pressure at the gas–liquid interface, are evaluated by a customized transmembrane pressure measurement (Supplementary Fig. S5). As shown in Fig. 3a, the gas must deform the gating liquid interface to enter into the substrate pores. Because of the strong solid–liquid interaction when UV light is off, the gas cannot open the liquid gating system under a small pressure difference (Δ*P*) between both sides of the porous substrate. Whereas during UV irradiation, the gas could easily pass through the porous substrate and create an open, liquid-lined pathway driven by Δ*P* due to the weak solid–liquid interaction. Further, as the capillarity reconfigures the pore-filling liquid rather than draining it away, the substrate pores would be reclosed as soon as the photoisomerization conversing.

**Fig. 3:**
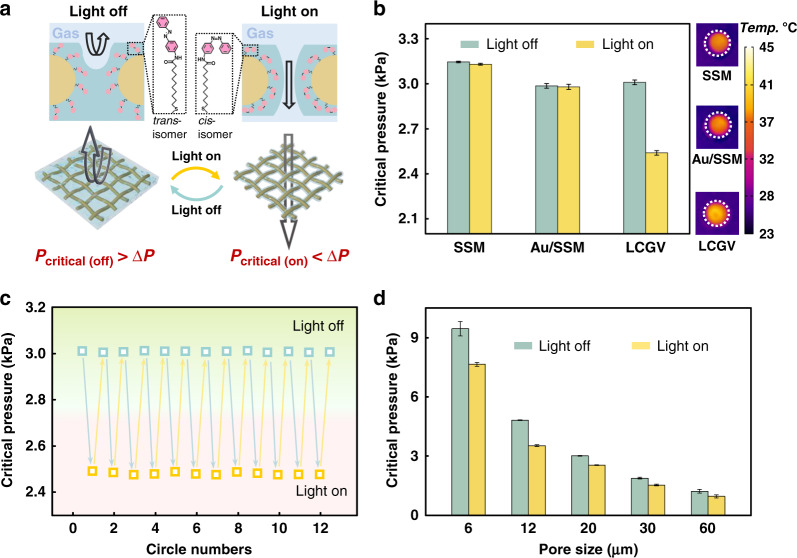
The non-thermal effective light-responsive gating performance of the LCGV system. **a** Schematic illustration of the molecular photoswitch photoisomerization and gating liquid reconfiguration for light-regulated gas transport. **b** Critical pressures of different liquid gating systems with or without UV irradiation, respectively (left). The infrared images and central temperature of different liquid gating membranes when transporting gas under UV irradiation (right). **c** Critical pressures for gas through LCGV system during alternative irradiation cycles. **d** Critical pressures for gas through LCGV systems with different substrate pore sizes

Figure 3b shows the required gating thresholds of gas transmembrane pressure for different liquid gating systems. Comparing with the non-responsive liquid-infused membranes (SSM and Au/SSM systems), the photoisomerization endows an LCGV system (Azo/SSM) with threshold variation of the substantial critical pressures (*P*_critical (on)_ and *P*_critical (off)_) of ~0.5 kPa under light stimuli.

To eliminate the influence on critical pressure caused by thermal-induced SFT changes^33^, a low-temperature narrow-band UV light source was used as the trigger for gas flow control and the temperature gradient of the UV irradiation area on liquid-infused membrane was measured by infrared (IR) thermal imaging. As shown in Fig. 3b right, the UV light does not cause a significant temperature rise in its irradiation area, which has ensured a stable SFT of the gating liquid used in the SSM and Au/SSM systems. Thus, the liquid gating systems without molecular photoswitch grafting show almost constant critical pressures and non-responsive liquid gating behavior under light stimuli, which further demonstrates the non-thermal liquid gating performance of the LCGV system. Moreover, LCGV system displays controllable and stable gating performance for azobenzene-based molecular photoswitches transport (Fig. 3c) and its working pressure range can be precisely modulated by manageably changing the pore sizes of porous substrate to satisfy different pressure environments (Fig. 3d).

Corrosion is one of the major reasons for the failure of metallic parts^18^, even the austenitic stainless steel, which is well known for its excellent corrosion resistance owing to the nano-scale oxide protective layer on surface, are often pitting corroded when exposed to the aggressive radicals, such as chloride radicals^34,35^. In this work, austenitic steel AISI 316 is used as the porous substrate of the LCGV system; therefore, the evaluation of corrosion resistance of the liquid gating system is of vital importance (Supplementary Fig. S7). In the corrosion test (Fig. 4a), the specific corrosive gas forced through the SSM and LCGV system for a certain time, respectively. In comparison with the rapid and violent corrosion of SSM, the LCGV system represents an excellent chemical corrosion resistance even after forcing through the corrosive gas for 1 h, owing to the gating liquid with chemical inertness preventing the corrosive gas from contacting with the metallic membrane. Thus, this LCGV system is expected to be applied as a safe and stably controllable gas-flow control valve.

**Fig. 4:**
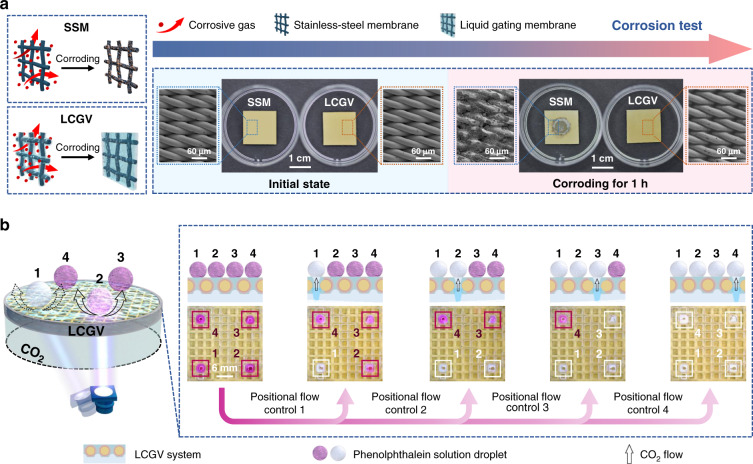
Corrosion resistance of the LCGV system and its light-regulated gas valve application with non-thermal effective gating-liquid positional flow control. **a** Corrosion tests of SSM and LCGV system. **b** Non-thermal effective light-regulated positional gas-triggered reaction under a steady-state pressure

For the application of LCGV system as a gas valve with non-thermal effective positional flow control, CO_2_ with a steady-state pressure was used as the transport gas to realize the gas-triggered reaction with high spatial and temporal resolution (Fig. 4b). In general, most of the gas-involved chemical reaction is accompanied by the applied pressure changes. However, the unstable pressure conditions may affect the reaction stability, yield, and even safety. The liquid gating gas valve with dynamically reconfigurable liquid gate can flexibly control the gas flow under a constant pressure, which ensures the stable, safe, and effective gas-involved reaction. At the initial state, alkaline phenolphthalein solution droplets (pH ~ 8.5) with red color were placed in different positions above the designated reaction device with the LCGV system (Fig. 4b left and Supplementary Fig. S8), of which the pores capillary-stabilizing gating liquid (*P*_critical (off)_) encapsulated CO_2_ in the chamber with a steady-state pressure (Δ*P*). With UV irradiation, the gas critical pressure decreased dramatically, owing to the solid–liquid adhesive interaction variation induced by the photoisomerization of molecular pohotoswitches at the selected position. When Δ*P* was higher than the substantial critical pressure (*P*_critical (on)_) under irradiation, CO_2_ penetrated through the LCGV system to trigger the neutralization reaction and the red solution tended to colorless gradually. By repositioning the UV light source, the previous photoisomerization reversed and replaced by the newly induced photoisomerization at designated position, and a new neutralization reaction occurred. Consequently, this LCGV with non-thermal effective liquid-gating positional flow control opens a new route for the contactless and precisely gas-involved reaction control in microfluidics.

To sum up, we have established a non-thermal effective light-responsive and corrosion-resistant liquid gating system with precisely position-specific gas flow control. Theoretical analysis and experimental data demonstrate the light-regulated gas-flow control valve constructed with this liquid gating system, which is achieved by a rational solid–liquid interface design. Owing to the solid surface polarity changes caused by the reversible photoisomerization of azobenzene-based molecular photoswitches grafted on porous substrate, the affinity between the gating liquid and solid porous substrate can be dynamically regulated by light stimuli, which further realizes the light-responsive dynamic gas flow control by the LCGV system. Meanwhile, the LCGV system possesses an excellent corrosion resistance due to the gating liquid protection. In addition, considering the athermal interaction, free localizability, and contactless and fast response of UV light, this LCGV achieves a precisely positional gas flow control without thermal interference under a steady-state pressure, which is expected to be used further in contactless and precisely gas-involved reaction control in microfluidics.

## Methods

### Chemicals

SSM (316 L, pore size of 20 μm) was purchased from Anping Tianhong Metal Mesh Factory. Ethanol, ethylene glycol, glycerol, tetrahydrofuran, hydrochloric acid, nitric acid, and phenolphthalein were purchased from Sinopharm Chemical Reagent Co., Ltd. 11-Mercaptoundecanoic acid, *p*-aminoazobenzene, diiodomethane, and silicone oil were purchased from Aladdin Industrial Co., Ltd. *N*-(3-dimethylaminopropyl)-*N*’-ethylcarbodiimide hydrochloride was purchased from Sigma-Aldrich. Air was used as the transport gas. Milli-Q water with a resistivity of 18.2 MΩ cm was used in all experiments. Liquid gating membranes were prepared by infusing the gating liquids into the porous substrates. Rhodamine B (RB) aqueous solution was prepared by dissolving RB powders into the Milli-Q water at a final concentration of 0.1 mg ml^−1^.

### Fabrication of the light-responsive membrane

First, the bare SSM (1 cm × 1 cm) was cleaned through ultrasonic in ethanol, water, and ethanol in sequence for 30 s, to obtain a clean surface, and then dried in an oven. After that, the clean bare membrane was treated by Au sputtering under 8 mA for 60 s using the ion sputter (SBC-12, KYKY Technology Co., Ltd, China). Then the Au-coated membrane was immersed into the ethanol solution containing 11-mercaptoundecanoic acid (10 mM) for 6 h at 40 °C, to obtain a carboxyl group on the surface of the membrane. After washing by ethanol and drying, the membrane was immersed into the ethanol solution containing *p*-aminoazobenzene (10 mM) and *N*-(3-dimethylaminopropyl)-*N*’-ethylcarbodiimide hydrochloride (10 mM, as the catalyst) for 6 h at 40 °C. In the presence of the catalyst, the azobenzene-based molecular photoswitches were grafted onto the membrane through the formation of an amine bond.

### The work of adhesion analysis

The SE of light-responsive surface and *W*_A_ between functional liquids and light-responsive surface under light stimuli were measured by the OWRK method on an OCA100 CA meter. Water, ethylene glycol, glycerol, and diiodomethane were used as reference liquids. The sessile drop and captive bubble methods were employed to evaluate the CAs of reference liquids on the light-responsive surfaces to calculate its SE under light stimuli. For the *W*_A_ calculation, the SFT with disperse *γ*^d^ and polar *γ*^p^ contributions of the reference liquids was obtained from the OCA100 software.

### Transmembrane pressure measurements

The gas pressure difference (Δ*P*) between both sides of the liquid gating system under light stimuli was measured with a self-designed set-up (Supplementary Fig. S5) by wet/wet current output differential pressure transmitters (PX273-020DI) from OMEGA Engineering, Inc. (Stamford, CT, USA). A flow rate of 2 ml min^−1^ given by Harvard Apparatus PHD ULTRA Syringe Pump was used in all the transmembrane pressure measurement experiments. UV spotlight (SP-9, Japan) with narrow-band wavelength around 365 nm and optical power density of 75.4 mW cm^−2^ was used to trigger the *trans*-to-*cis* photoisomerization and the mild indoor visible light with optical power density of 0.4 mW cm^−2^ was used to reverse the photoisomerization in all experiments. The irradiation time is 60 s. The response time for the light-responsive liquid gating system to open and close is about 1.4 s and 0.2 s, respectively. The indoor temperature is 21 °C and the relative humidity is 61% during the measurement.

### Anti-corrosion test

For the gaseous corrosion, nitrohydrochloric acid, the hydrochloride acid, and nitric acid solution with a volume ratio of 3 : 1 was boiled to obtain the corrosive gas that was used. The corrosive gas forced through the SSM (as a control) and LCGV system, respectively, with a flow rate of 200 μl min^−1^ for 1 h to observe the corrosion phenomena.

In addition, for the liquid corrosion, the glycerol, hydrochloride acid, and nitric acid solution with volume ratio of 2 : 3 : 1 was used as the corrosive solution. The corrosion solution (1 ml) was dropped onto the SSM (as a control) and LCGV system, to observe the corrosion phenomena of the membranes.

### Precisely positional gas-flow control application with the LCGV system

The self-designed device (Supplementary Fig. S8) with the CO_2_ as transport gas was used for the positional gas-flow control application. The LCGV system was sealed in the device as light-responsive gas valve. CO_2_ was encapsulated in the transparent chamber at the bottom of the device with a constant pressure of ~2.8 kPa. Four droplets of red alkaline phenolphthalein solution (pH ~ 8.5) were placed on the top of the device with different positions. UV spotlight was applied to trigger the positional neutralization reaction by repositioning the light source. Under UV irradiation, CO_2_ penetrated through the LCGV system in the selected area and the neutralization reaction occurred, resulting in changes of droplets in color.

### Characterizations

The morphology of the solid porous substrate was characterized by the high-resolution field-emission scanning electron microscopy (Zeiss, GeminiSEM 500, Germany). The element distributions on the solid porous substrate were obtained by energy-dispersive spectrometer (Zeiss, GeminiSEM 500, Germany). The photoisomerization of molecular photoswitches was measured using UV-visible near-IR spectrophotometer (PerkinElmer, Lambda 1050+, USA). The Fourier transform IR (FT-IR) analysis was carried out on in-situ FT-IR spectrometer (Bruker Vertex 70V, Germany) using KBr pellets in the range of 400–4000 cm^−1^. The wettability of different liquids on light-responsive surfaces under light stimuli was measured by sessile drop and captive bubble methods on the CA meter (DataPhysics, OCA100, Germany). The liquid droplets or gas bubble of 3 μL were placed in different areas on the surface. The value of CA was tested with an average of three independent measurements at least. Photographs and movies were taken by the camera (Nikon, D5000, Japan). The fluorescence images were obtained on the biological microscope (OLYMPUS, IX73, Japan).

## Supplementary information

Supplementaty information

Infrared thermal imaging for SSM

Infrared thermal imaging for AuSSM

Infrared thermal imaging for AzoSSM

Liquid corrosion test

Positional gas flow control

## Data Availability

The supporting data for the findings in this study are available from the corresponding author upon reasonable request.
