# Supplementary material for: Light-responsive and corrosion-resistant gas valve with non-thermal effective liquid-gating positional flow control
Source: Light Sci Appl. 2021 Jun 16;10:127. doi: 10.1038/s41377-021-00568-9 (PMC8209104; doi:10.1038/s41377-021-00568-9)
Supplement: Supplementary file 1 — Supplementaty information [file 41377_2021_568_MOESM1_ESM.docx]

**Supplementary Information for**

**Light-responsive and corrosion resistant gas valve with non-thermal effective liquid gating positional flow control**

Baiyi Chen^1,4†^, Rongrong Zhang^1†^, Yaqi Hou^1^, Jian Zhang^1^, Shiyan Chen^1,4^, Yuhang Han^1^, Xinyu Chen^1^ and Xu Hou^1,2,3,4,5^*

^1^State Key Laboratory of Physical Chemistry of Solid Surfaces, College of Chemistry and Chemical Engineering, Xiamen University, Xiamen 361005, China

^2^Department of Physics, Research Institute for Biomimetics and Soft Matter, Fujian Provincial Key Laboratory for Soft Functional Materials Research, Jiujiang Research Institute, College of Physical Science and Technology, Xiamen University, Xiamen 361005, China

^3^ College of Materials, Xiamen University, Xiamen 361005, China.

^4^Collaborative Innovation Centre of Chemistry for Energy Materials, Xiamen University, Xiamen 361005, China

^5^Tan Kah Kee Innovation Laboratory, Xiamen 361102, China

^†^These authors contribute equally to this work.

*Corresponding author. Email: [houx@xmu.edu.cn](mailto:houx@xmu.edu.cn) Tel: +86 592 2180937

Email: Baiyi Chen ([baiyichen@xmu.edu.cn](mailto:baiyichen@xmu.edu.cn)); Rongrong Zhang ([zhangrr@stu.xmu.edu.cn](mailto:zhangrr@stu.xmu.edu.cn));

Yaqi Hou ([houyq@xmu.edu.cn](mailto:houyq@xmu.edu.cn)); Jian Zhang ([jianzhangchem@stu.xmu.edu.cn](mailto:jianzhangchem@stu.xmu.edu.cn));

Shiyan Chen (sychen@stu.xmu.edu.cn); Yuhang Han ([hanyh@stu.xmu.edu.cn](mailto:hanyh@stu.xmu.edu.cn));

Xinyu Chen ([xy.chen@xmu.edu.cn](mailto:xy.chen@xmu.edu.cn))

*Dedicated to the 100^th^ Anniversary of Xiamen University*

**S1 Demonstration of the molecular photoswitches on membrane**


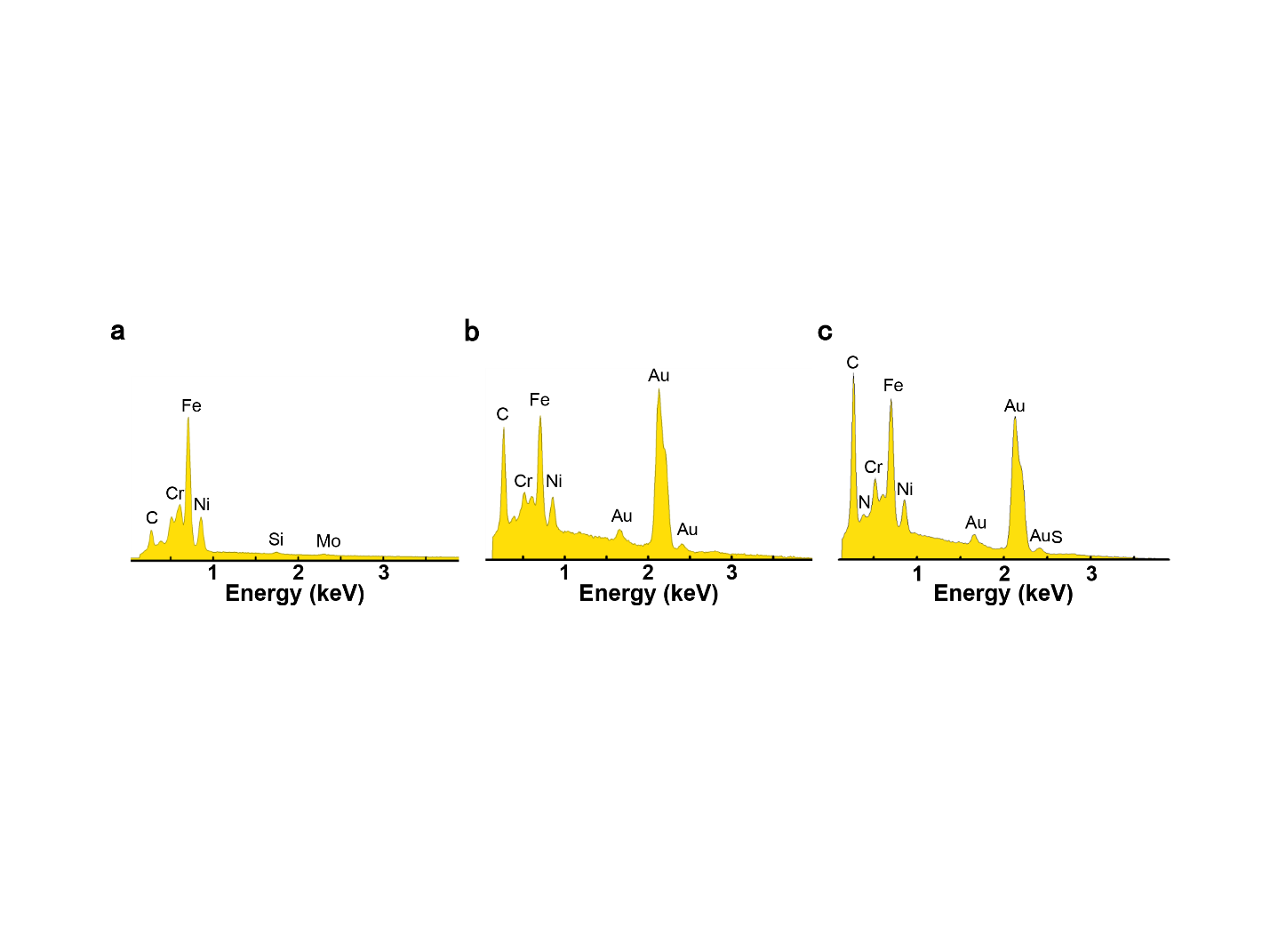


**Fig. S1: Elemental analysis of different membranes by energy dispersive spectrometers.** The element analysis of (**a**) SSM, (**b**) Au/SSM and (**c**) Azo/SSM. SSM, Au/SSM and Azo/SSM represent the bare stainless-steel membrane, Au-ion sputtered stainless-steel membrane and azobenzene-based molecular photoswitches grafted stainless-steel membrane. As can be seen, after modification of the azobenzene-based molecular photoswitches, nitrogen and sulfur element belong to the photoswitches can be found in Fig. S1c, which demonstrates that the azobenzene-based molecular photoswitches were successfully modified on the stainless-steel membrane.

**S2 Synthesis of the molecular photoswitch and its fast reversible photoisomeriazation**

The molecular photoswitch was obtained by one-step method from 11-mercaptoundecanoic acid and *p*-aminoazobenzene, and its synthesis was proved by infrared spectrum analysis (Fig. 2b). In the FT-IR spectra of 11-mercaptoundecanoic acid, the peaks at 2920 cm^-1^ and 2850 cm^-1^ belong to the stretching vibration of C-H bonds, and the stretching vibration peak at 1702 cm^-1^ belong to the C=O bond of carboxyl group.^1^ In the FT-IR spectra of *p*-aminoazobenzene, the stretching vibration peaks at 3469 cm^-1^ and 3368 cm^-1^ (intermolecular) belong to the N-H bond of amino group, and the peak at 1414 cm^-1^ corresponds to the stretching vibration of the azo group. The peaks at 1606 cm^-1^ and 1000~1300 cm^-1^ belong to the C-C bonds stretching vibration and the fingerprint of the benzene ring, respectively.^2,3^ In the FT-IR spectra of molecular photoswitch, the peaks at 2920 cm^-1^ and 2850 cm^-1^ correspond to the stretching vibration of C-H bonds of the alkane chain, and the stretching vibration peak of azo group is located at 1396 cm^-1^. The C-C bonds stretching vibration peak and the fingerprint of the benzene ring are witnessed at 1602 cm^-1^ and 1000~1300 cm^-1^, respectively.^4^ All the mentioned above peaks of molecular photoswitch correspond to the FT-IR spectra of 11-mercaptoundecanoic acid and *p*-aminoazobenzene. Additionally, the peaks at 1670 cm^-1^ and 1535 cm^-1^ belong to the amide I band and amide II band respectively and the peak at 3326 cm^-1^ attributes to the stretching vibration of N-H bond of amide group, which demonstrates the successfully synthesis of molecular photoswitch from 11-mercaptoundecanoic acid and *p*-aminoazobenzene.


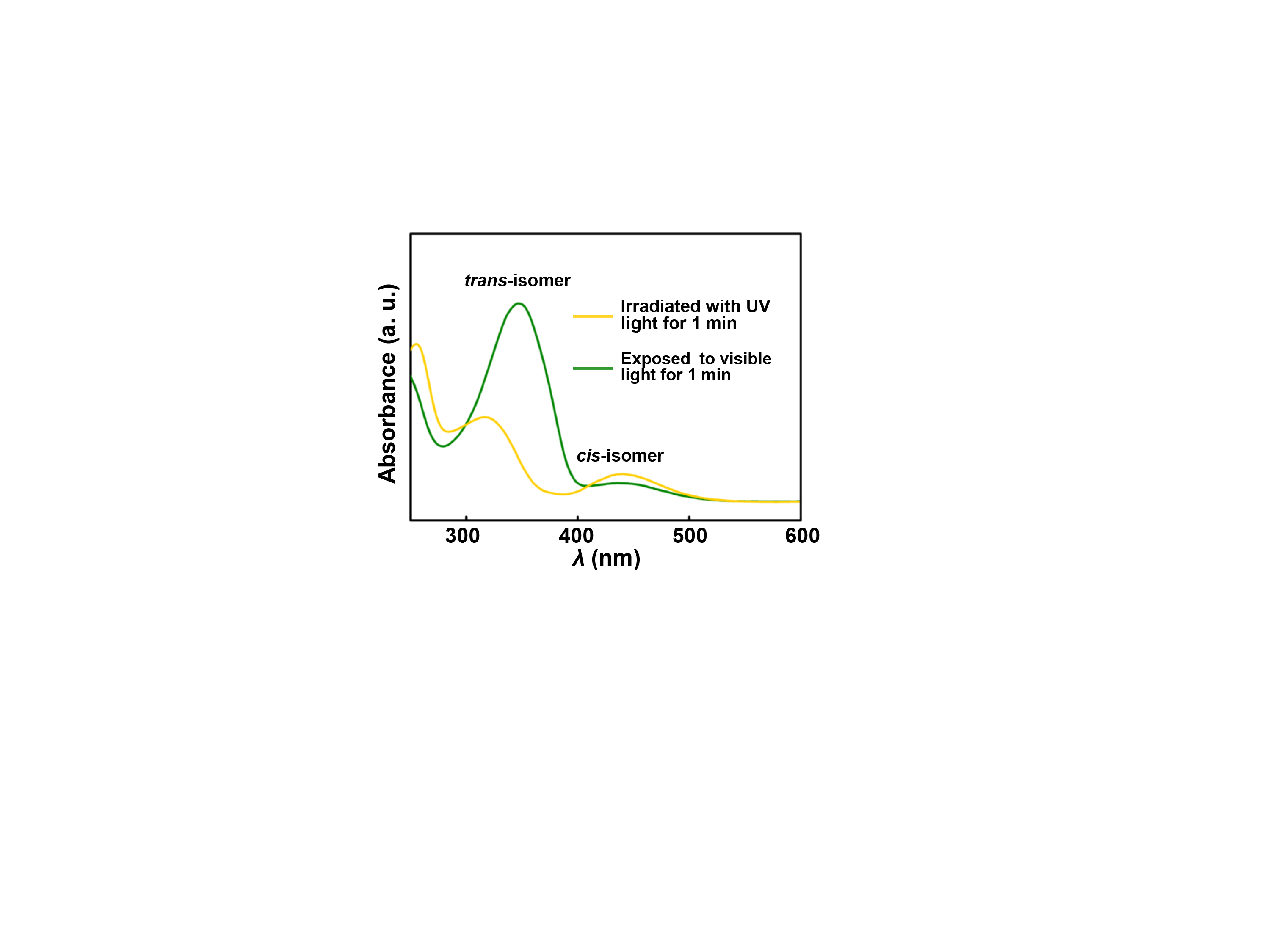


**Fig. S2: UV-vis absorption spectra of the azobenzene-based molecular photoswitch with *trans* and *cis* forms.** The molecular photoswitch undergoes a *trans*-to-*cis* photoisomerization by UV irradiating for 1 min and convers to *cis* form after exposing to indoor visible light for 1 min, indicating a fast light responsiveness.

**S3 Wettability changes and the work of adhesion analysis of LCGV system under light stimuli**


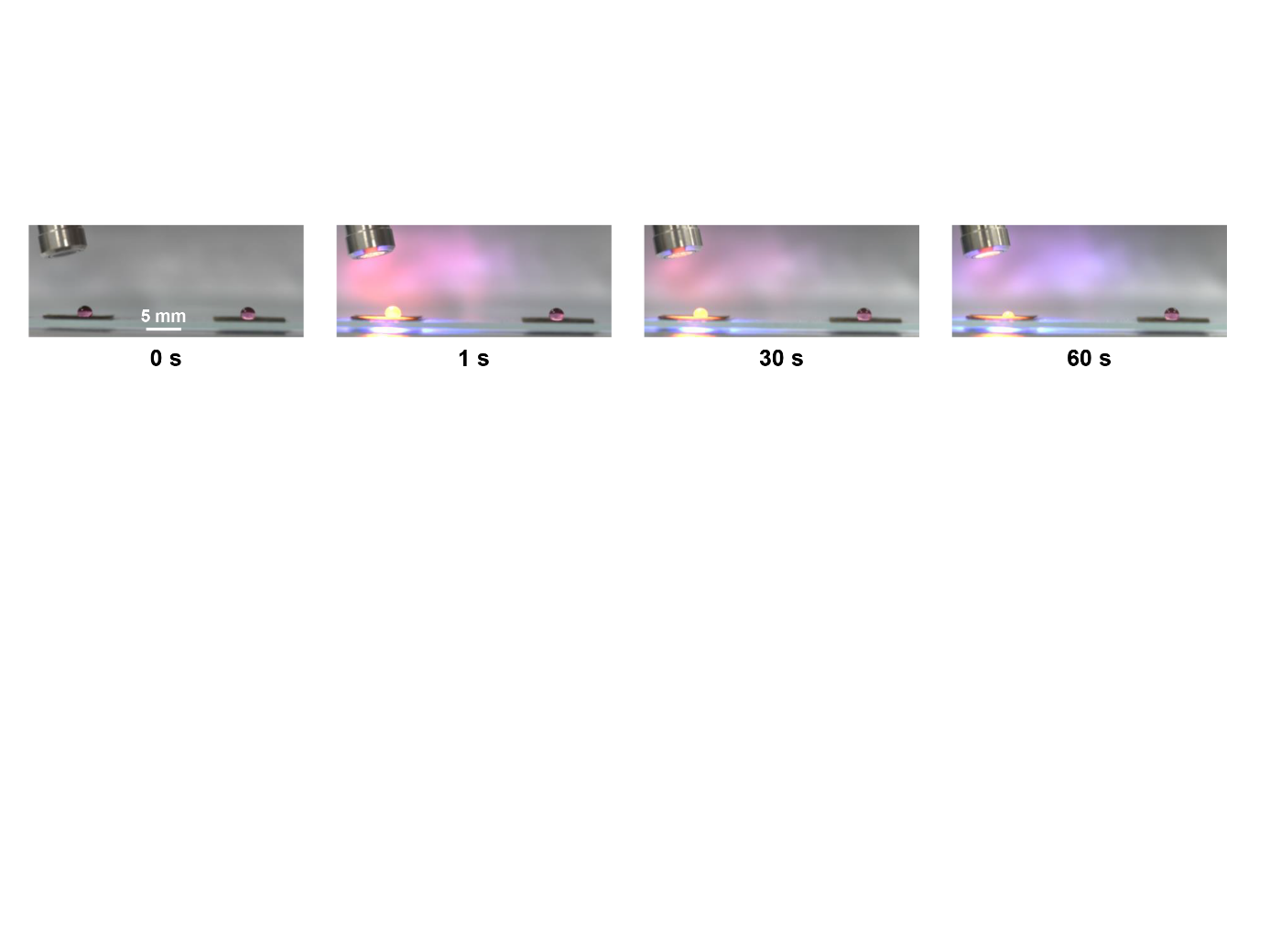


**Fig. S3: Photographs of the changes in water droplets wettability on Azo/SSM with or without UV irradiation.** 3 μL of water droplets were dropped onto the surfaces of two same Azo/SSMs. With UV irradiation (left), the water droplet was gradually adsorbed by the Azo/SSM, indicating a wettability change towards hydrophilicity of this molecular photoswitch grafted membrane. While water droplet without UV irradiation remain constant (right).

Liquids exert an adhesive interaction with their contacting solids. If we want to analyze the adhesive interaction between liquids and solids, it is necessary to know the optimum work of adhesion of the liquid on the chosen surface. Owens-Wendt-Rabel-Kälble (OWRK) method was used to calculate the work of adhesion.

$W_{A}= \sigma_{l}\left( 1+\cos\theta\right)$ (1)

where $W_{A}$ is the work of adhesion, $\sigma_{l}$ is the surface tension of liquid and $\theta$ is the contact angle.

According to OWRK method, the work of adhesion can be shown as the following equation:

$W_{A}=2(\sqrt{\sigma_{l}^{d}.\sigma_{s}^{d}}+\sqrt{\sigma_{l}^{p}.\sigma_{s}^{p}})$ (2)

where $\sigma_{s}$ is the surface energy of solid. $\sigma_{s}^{d}$ and $\sigma_{s}^{p}$ are the dispersive contribution and polar contribution of the solid surface energy respectively. $\sigma_{l}^{d}$ and $\sigma_{l}^{p}$ are the dispersive contribution and polar contribution of the liquid surface tension respectively.

The surface tension of liquids $\sigma_{l}$ can be shown as following:

$\sigma_{l}=\sigma_{l}^{d}+\sigma_{l}^{p}$ (3)

From equations (2) and (3) we can obtain the work of adhesion:

$W_{A}=2(\sqrt{\left( \sigma_{l}-\sigma_{l}^{p} \right).\sigma_{s}^{d}}+\sqrt{\sigma_{l}^{p}.\sigma_{s}^{p}})$ (4)

For a certain solid surface,$\sigma_{s}^{p}$ and $\sigma_{s}^{d}$ are constant, for a constant work of adhesion, a function $\sigma_{l}\left( \sigma_{l}^{p} \right)$ for $\sigma_{l}^{p}\geq0$.

$\sigma_{l}\left( \sigma_{l}^{p} \right)=\frac{\left( \frac{W_{A}}{2}-\sqrt{\sigma_{s}^{p}.\sigma_{l}^{p}} \right)^{2}+\sigma_{s}^{d}.\sigma_{l}^{p}}{\sigma_{s}^{d}}$ (5)

This multi parameter equation (5) can be used to provide the ‘isograms’ of $W_{A}$ between liquids and solids. Each work isogram includes a minimum, describing the lowest surface tension, for a defined polar contribution that corresponds to that level of work. A liquid with these parameters will exert a maximum adhesive force on the given surface.

If the derivative (5) is set = 0, the polarity of the minimum can be calculated. For $\sigma_{l_{min}}^{p}$ the following is valid:

$\sigma_{l_{min}}^{p}=\left( \frac{W_{A}\sqrt{\sigma_{s}^{p}}}{2\left( \sigma_{s}^{p}+\sigma_{s}^{d} \right)} \right)^{2}$ (6)

For each level of the $W_{A}$, a liquid with identified polarity and relevant surface tension will exert optimum adhesion. A straight line can be plotted through these optimum adhesive force for each level of work and arrange of acceptable performance (+/- % of optimum line) proposed.


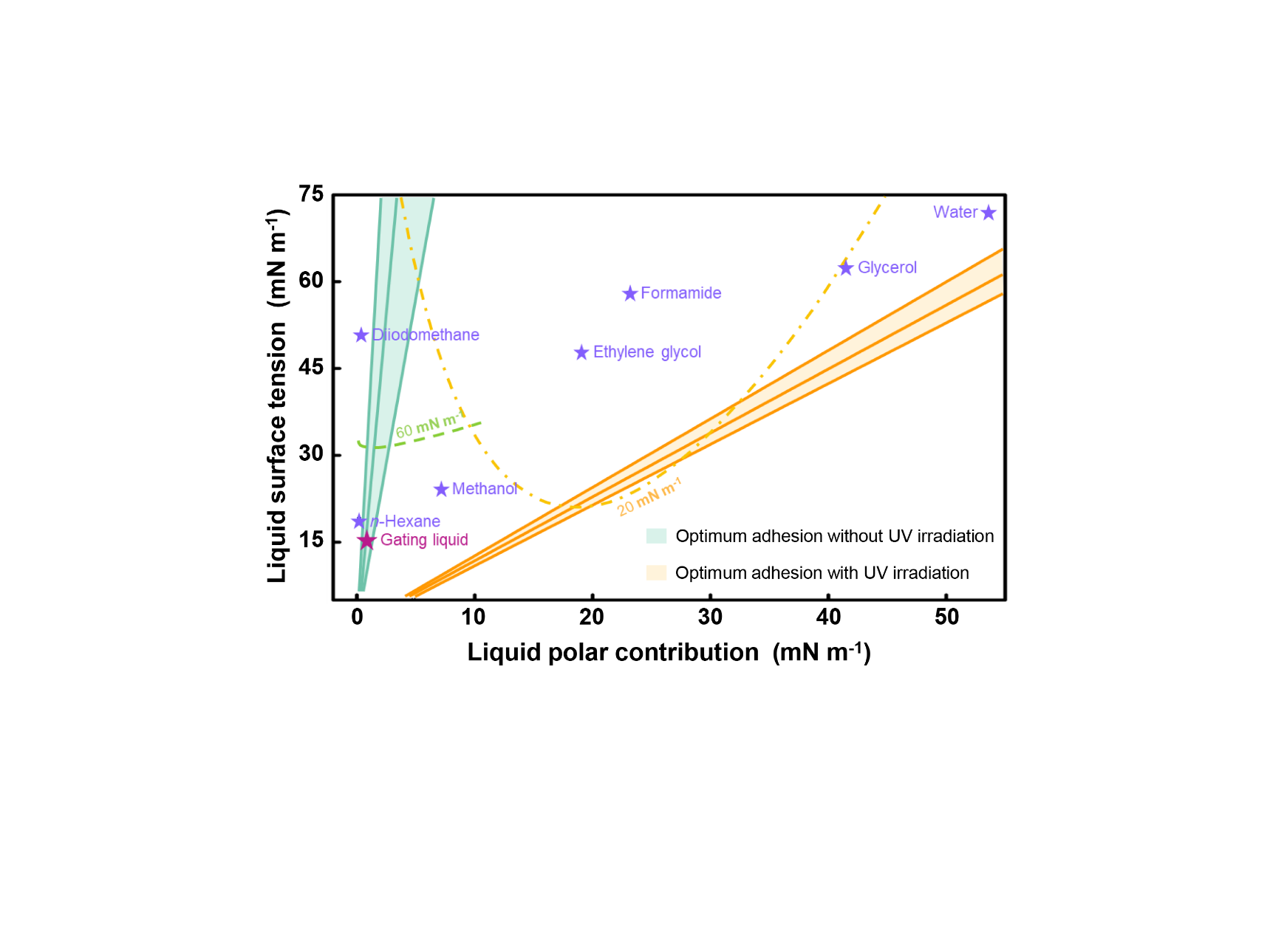


**Fig. S4: The selection of gating liquid based on the work of adhesion analysis.** A series of functional liquids with different surface tension and polarity have been used for the work of adhesion analysis to select the gating liquid. As can be seen in this figure, the nonpolar functional liquid of Krytox® 103 presented an optimum adhesion performance with the chosen light-responsive surface under light stimuli, which showed a strong adhesive interaction with solid surface without UV irradiation (being in the corridor for optimum adhesion without UV irradiation) and a weak adhesive interaction with solid surface under UV irradiation (being out the corridor for optimum adhesion with UV irradiation). This phenomenon would lead to a variation of substantial critical pressure for gas transport with LCGV system under light stimuli.

**S4 The light-responsive gating performance for gas transport of LCGV system**


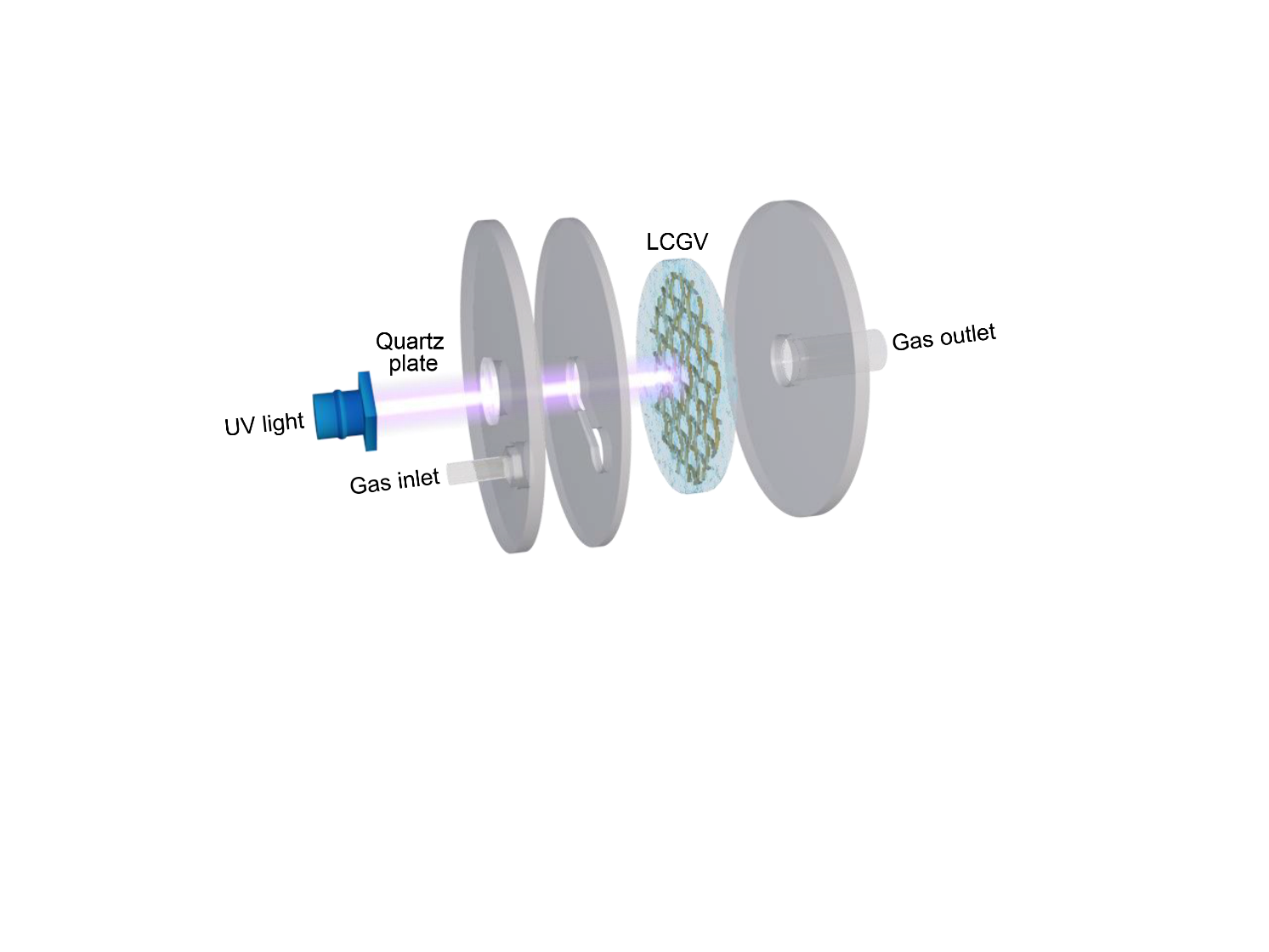


**Fig. S5: The schematic diagram of self-designed set-up for transmembrane pressure measurement with LCGV system.**

**S5 Anti-fouling and liquid corrosion-resistent tests of LCGV system and its light-regulated gas valve application with non-thermal effective gating liquid positional flow control**


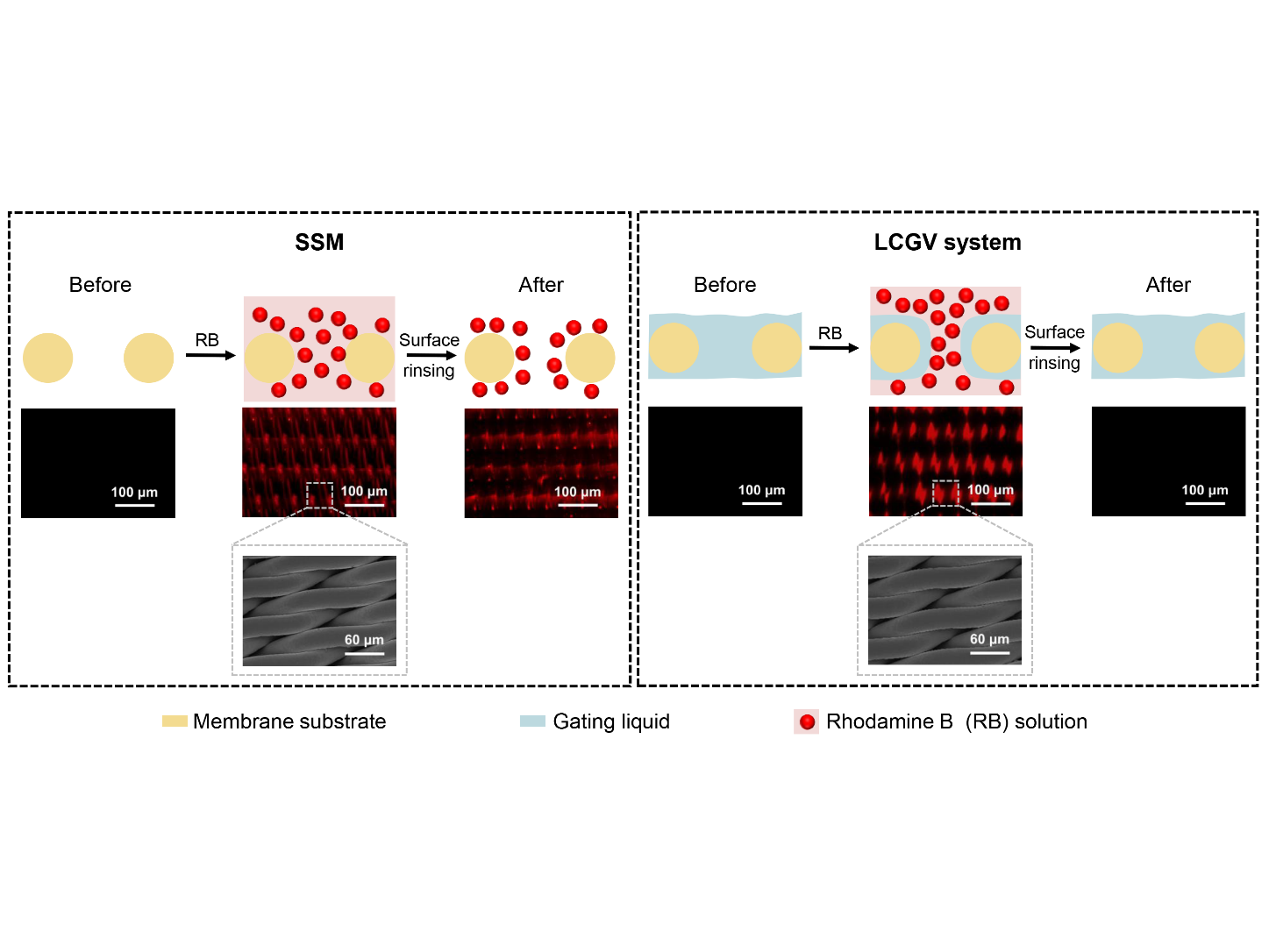


**Fig. S6: Anti-fouling measurements of (a) SSM and (b) LCGV system.** The figures show the schematic diagrams (top), the fluorescence and SEM images (bottom) of the anti-fouling measurements for bare stainless-steel membrane and LCGV system. As can be seen in this figure, after penetrating through the rhodamine B solution, both bare stainless-steel membrane and LCGV system were completely contaminated. Then, they were fully washed with Milli-Q water, LCGV system recovered to the initial state without any contamination, while bare stainless-steel membrane was still contaminated. This is because that the gating liquid possesses a low adhesion with rhodamine B solution and prevents it from contacting the membrane substrate, ultimately indicating an anti-fouling property of LCGV system.


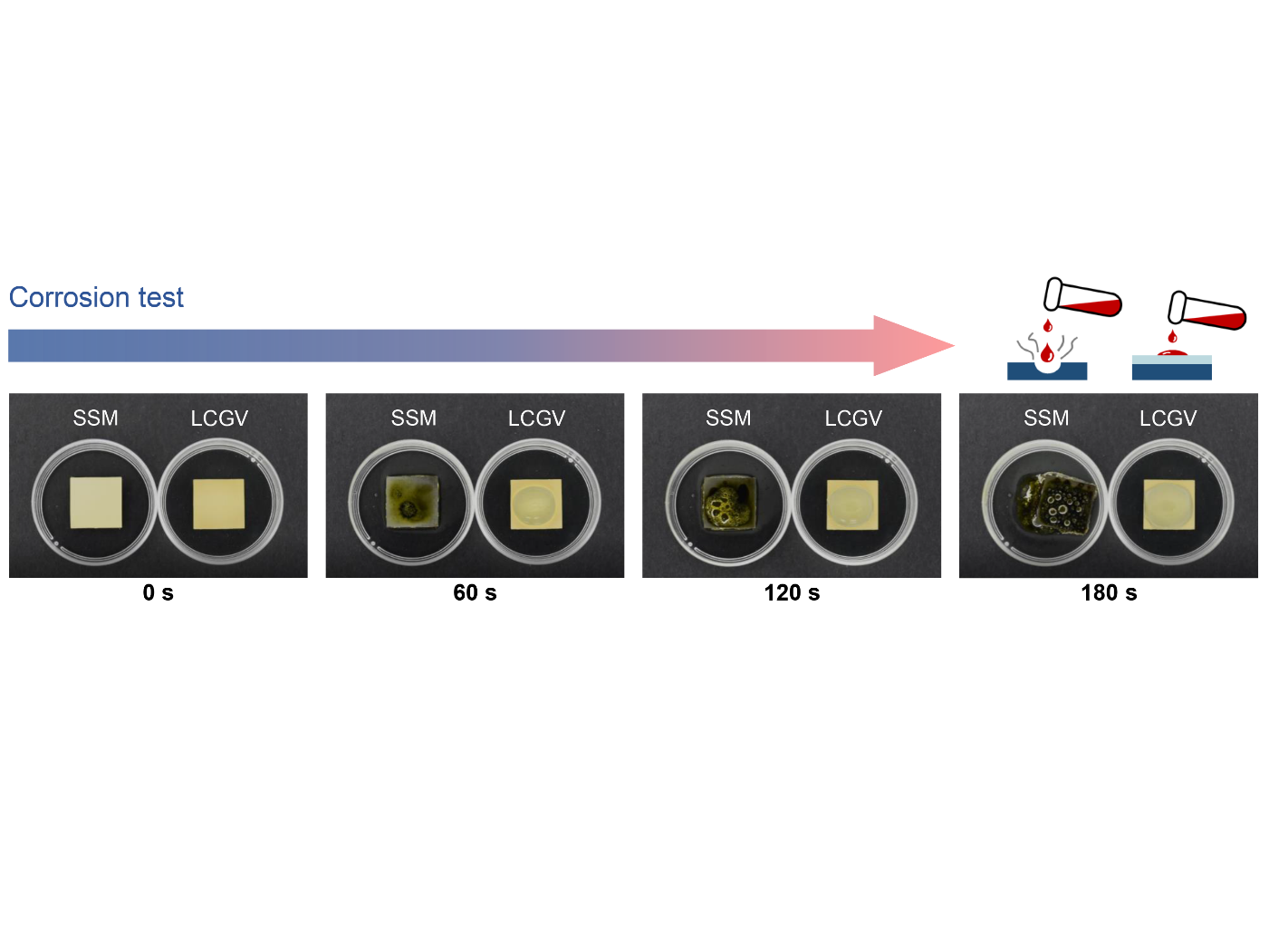


**Fig. S7: Liquid corrosion-resistant test of SSM and LCGV system.** Comparing with the serious corrosion of bare stainless-steel membrane, the LCGV system remains a good corrosion resistance even after contacting with the corrosive solution for 180 s.


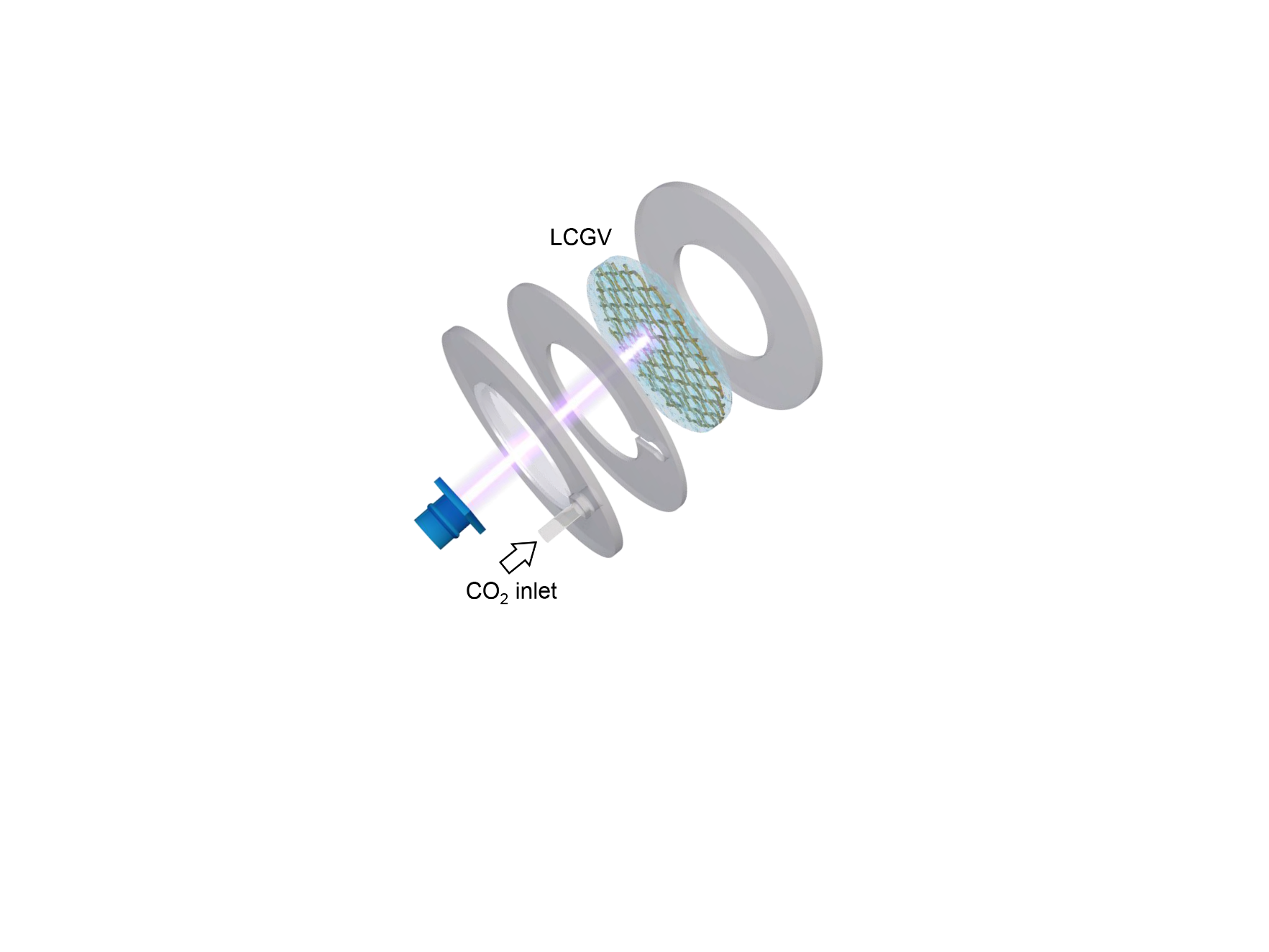


**Fig. S8: The schematic diagram of self-designed set-up for positional gas flow control application with LCGV system.**

**References**

1. Dana, W. *et al*. Course notes on the interpreatation of infrared and raman spectra (John Wiley & Sons, Inc. 2003).
2. Ya, Q. *et al.* The synthesis of aminoazobenzenes and the effect of intermolecular hydrogen bonding on their photoisomerization. *Dyes and Pigments.* **79**, 159-165 (2008).
3. Eazhilarasi, G. *et al*. Studies on crystal growth, vibrational and optical properties of organic nonlinear optical crystal: p-aminoazobenzene. *Spectrochimica Acta Part A: Molecular and Biomolecular Spectroscopy* **71**, 502-507 (2008).
4. Khan, A. *et al*. Synthesis of P(FHEMA-co-MAZO-co-MAA)s copolymers and their redox and photo-responsive properties. *Journal of Organometallic Chemistry* **902**, 120955 (2019).
